# Regulation of DNA Damage Response by Estrogen Receptor β-Mediated Inhibition of Breast Cancer Associated Gene 2

**DOI:** 10.3390/biomedicines3020182

**Published:** 2015-04-21

**Authors:** Yuan-Hao Lee, Youping Sun, Leo E. Gerweck, Randolph D. Glickman

**Affiliations:** 1 Department of Radiology, University of Texas Health Science Center at San Antonio, San Antonio, TX 78229, USA; 2 Department of Radiation Oncology, Center for Radiological Research, Columbia University Medical Center, New York, NY 10032, USA; E-Mail: ys2611@cumc.columbia.edu; 3 Department of Radiation Oncology, Massachusetts General Hospital, Boston, MA 02114, USA; E-Mail: lgerweck@mgh.harvard.edu; 4 Department of Ophthalmology, Center for Biomedical Neuroscience, University of Texas Health Science Center at San Antonio, San Antonio, TX 78229, USA; E-mail: Glickman@uthscsa.edu

**Keywords:** BCA2, DNA damage response, Erb-041, Rad51, UVC

## Abstract

Accumulating evidence suggests that ubiquitin E3 ligases are involved in cancer development as their mutations correlate with genomic instability and genetic susceptibility to cancer. Despite significant findings of cancer-driving mutations in the *BRCA1* gene, estrogen receptor (ER)-positive breast cancers progress upon treatment with DNA damaging-cytotoxic therapies. In order to understand the underlying mechanism by which ER-positive breast cancer cells develop resistance to DNA damaging agents, we employed an estrogen receptor agonist, Erb-041, to increase the activity of ERβ and negatively regulate the expression and function of the estrogen receptor α (ERα) in MCF-7 breast cancer cells. Upon Erb-041-mediated ERα down-regulation, the transcription of an ERα downstream effector, *BCA2* (Breast Cancer Associated gene 2), correspondingly decreased. The ubiquitination of chromatin-bound BCA2 was induced by ultraviolet C (UVC) irradiation but suppressed by Erb-041 pretreatment, resulting in a blunted DNA damage response. Upon BCA2 silencing, DNA double-stranded breaks increased with Rad51 up-regulation and ataxia telangiectasia mutated (ATM) activation. Mechanistically, UV-induced BCA2 ubiquitination and chromatin binding were found to promote DNA damage response and repair via the interaction of BCA2 with ATM, γH2AX and Rad51. Taken together, this study suggests that Erb-041 potentiates BCA2 dissociation from chromatin and co-localization with Rad51, resulting in inhibition of homologous recombination repair.

## 1. Introduction

Breast cancer is the most frequently diagnosed site-specific cancer in the female population in the world. Its mortality rate has fallen with earlier diagnosis, and improvements in medical treatments, including surgery, chemotherapy, radiotherapy and anti-hormonal treatment. By blocking estrogen receptors (ER), the proliferation and metastatic potential of early stage breast cancers can be inhibited by aromatase inhibitors and selective ER modulators, such as Arimidex, Aromasin, Tamoxifen and Faslodex [1]. The reduced production of estrogen as well as competitive drug binding with ERα (estrogen receptor α) prevents ER−estrogen complexes from forming and translocating to the nuclei to trigger the transcription of growth-promoting genes. As ERα transactivates genes that promote accelerated DNA synthesis and cell proliferation, high levels of estrogens also increase DNA damage and the risk of breast and ovarian cancer. Nevertheless, estrogen also promotes the transcription of BRCA1 via binding with an ERα/p300 complex, and facilitates DNA double-stranded break (DSB) repair by stimulating the formation of a complex among ERα, CREB-binding protein and BRCA1 [2]. It has been shown that the ubiquitin E3 ligase activity of BRCA1 is critical for homologous recombination (HR) repair [3]. With respect to the high propensity of BRCA mutations in familial breast cancer, the facilitative role of BRCA1 in DNA damage response and repair is thought to be facilitated by other breast cancer-associated ubiquitin E3 ligases. To find out whether the ERα downstream effector, *BCA2* (breast cancer-associated gene 2), plays a role in DNA damage response (DDR) secondary to increased replication stress upon ERα-enhanced DNA synthesis, Erb-041 (an agonist of ERβ) was applied to inhibit ERα transcription activity prior to UVC irradiation [4,5,6]. The present study particularly demonstrated the mechanism of Erb-041 action in increasing carcinogen-induced DNA damage via the potentiation of BCA2 destabilization and the interaction between BCA2 and DDR proteins.

Activation of ERβ has proved to be therapeutically valuable for inhibiting ERα-mediated cell proliferation through the enhancement of ERα/β heterodimerization [7,8]. Among many downstream effecters of ERα, breast cancer-associated gene 2 (BCA2) was found to be trans-activatable by ERα and correlated with clinical variables, such as lymph node status and regional recurrence [9,10]. The correlation between the nuclear expression of BCA2 and positive ER status suggests that BCA2 may be involved in the adaptation of estrogen-responsive cancers to chronic replication stress by upregulating the cells’ DNA repair capability [11]. BCA2 has been characterized as an ubiquitin E3 ligase, RING-finger protein (RNF115), or Rab7-interacting RING-finger protein (Rabring7) that is overexpressed in more than 50 percent of breast tumors, including ER-negative breast cancers [12]. It is known that BCA2 promotes breast cancer development in association with ubiquitin-mediated degradation of p21^Waf1/Cip1^ via its E3 ubiquitin ligase activity [13]. In addition, BCA2 was found to complex with Rab7 (a cytosolic GTPase) and inhibit cellular endocytosis and lysosomal degradation of EGF, leading to EGF stabilization and enhanced cell proliferation [14,15]. However, it is unclear whether BCA2 plays a role in DNA damage response (DDR) to increased replication stress associated with enhanced cell proliferation, or in response to exogenous DNA damaging agents such as UV and X-rays.

Here, we assess the efficacy of an ERβ agonist as a DNA damage sensitizer in human breast cancer cells, using ultraviolet C (UVC) irradiation as an inducer of DNA damage. Compared with cisplatin, doxorubicin or X-rays, UVC induces various types of DNA damage, enabling the exploration of the effect of Erb-041 on multiple DNA repair pathways, such as ICL (interstrand crosslink) repair, homologous recombination repair, non-homologous end joining repair, and base and nucleotide excision repair. Based on the findings that the level of Rad51 mRNA is positively correlated to the status of estrogen receptors, and that ERβ inhibits homology-directed DNA repair by facilitating nuclear interaction between Rad51 and insulin receptor substrate 1 (IRS-1) in ERα-low-expressing medulloblastoma, we hypothesize that Erb-041 may potentiate UVC-induced DNA DSBs through HR inhibition [16,17,18]. In HR-directed DNA repair, Rad51 is loaded onto the 3’ ends of DNA DSBs for directing a template strand of DNA to a paired strand of homologous DNA molecules [19]. With assistance from its cofactors, Rad51 forms a helical nucleoprotein filament on DNA to elicit DNA strand exchange activity [20,21,22]. Given that IRS-1 binds ERα, translocates to the nucleus, and modulates ERα-dependent transcription at estrogen response elements (ERE), the inhibitory effect of ERβ on the transcription activity of ERα may further decrease cell survival via Rad51 inhibition [23]. The synthetic ERβ agonist, Erb-041, displays more than a 200-fold greater selectivity for ERβ *vs.* ERα [24]. The agonistic effect of Erb-041 on ERβ was found to increase the growth inhibitory effect of Tamoxifen in the combinatory treatment of MCF-7 and T-47D cells [25]. Well-tolerated with few side effects, ERβ agonists are currently utilized in clinical trials for the treatment of patients with cancer and other inflammatory diseases [26,27]. With the encouraging cancer-suppressing feature of Erb-041, we herein describe its *in vitro* anticancer activity via the modulation of DNA damage response and repair as well as its counteractive action on the ERα-BCA2 pathway.

## 2. Experimental Section

### 2.1. Cell Culture

MCF-7 and HEK293T/17 cells were maintained in DMEM/F12 (Life Technologies, Grand Island, NY, USA) and DMEM (Mediatech Inc., Manassas, VA, USA), respectively. Culture media were supplemented with 10% (*v*/*v*) fetal bovine serum (Atlanta Biologicals, Lawrenceville, GA, USA), 50 U/mL penicillin and 50 μg/mL streptomycin (Life Technologies) in 5% CO_2_ at 37 °C in a humidified incubator. Prior to addition of siRNA, the complete DMEM/F12 was replaced by reduced serum medium Opti-MEM (Life Technologies).

### 2.2. Cell Irradiation

Cells were exposed to UVC (XL-1000 UV crosslinker, Spectronics Corporation, Westbury, NY, USA) at the energy irradiance of 10 J/m^2^ after the culture media were replaced by phosphate-buffered saline (PBS) (Mediatech, Inc., Manassas, VA, USA). Cells were incubated with complete culture media immediately following cell irradiation.

### 2.3. Antibodies and Drugs

Anti-BCA2 antibody was kindly acquired from Dr. Arun Seth [11]. Antibodies against FANCD2 (sc-20022), Rad51 (sc-8349), β Tubulin (sc-9104) and actin (sc-8432) were purchased from Santa Cruz (Dallas, TX, USA); histone H2A (ab15653), lamin B1 (ab90576) were purchased from Abcam (Cambridge, MA, USA); Phospho-^1981^Ser-ATM (#4526), phospho-^139^Ser-γH2AX (#2577) and phospho-^317^Ser-Chk1 (#12302) were purchased from Cell Signaling (Beverly, MA); anti-mouse IgG-HRP (A9044), anti-goat IgG-HRP (A5420), anti-rabbit IgG-HRP (A9169), and FLAG M2 (F3165) were purchased from Sigma (St. Louis, MO, USA); Alexa Fluor 555 donkey anti-goat IgG (A-21432), Alexa Fluor 488 goat anti-mouse (A-10667) and donkey anti-rabbit IgG (A-21206) were purchased from Life Technologies. Erb-041 (chemical name: 7-Ethenyl-2-(3-fluoro-4-hydroxyphenyl)-5-benzoxazolol), acquired from Dr. Mohammad Athar, was dissolved in ethanol to 40 mM [4]. MG-132 (Selleck Chemicals, Houston, TX, USA) was dissolved in DMSO to 20 mM. Further dilution of the stock solutions by culture media was performed for cell treatment.

### 2.4. Plasmids, siRNAs and Transfection

Full-length human BCA2 cDNA, provided by Dr. Arun Seth, was cloned as a Not I–Sal I fragment from FLAG-tagged pCMV–BCA2 [11]. The vector, pcDNA3-EGFP (plasmid #13031), was acquired from Addgene (Cambridge, MA, USA). The siRNAs targeting the BCA2 gene were synthesized by Thermo Scientific Dharmacon (Lafayette, CO, USA), and the sequences were 5'-AGACAAUACCACAACAACATT-3' and 5'-CGUCUGAAUAGAAUUAAUUTT-3', respectively. A scrambled siRNA sequence, 5'-UAGCGACUAAACCACAUCAAUU-3', was used as the negative control. Transfections were performed using Lipofectamine 2000 (Invitrogen, Grand Island, NY, USA) with protocols recommended by the manufacturer.

### 2.5. Protein Extraction and Sodium Dodecyl Sulfate−Polyacrylamide Gel Electrophoresis (SDS-PAGE)

Cells were harvested by aspirating the medium and washing twice with cold PBS. Cell lysis was accomplished with cytoskeleton (CSK) buffer containing 0.1 M NaCl, 10 mM PIPES (pH 6.8), 3 mM MgCl_2_, 1 mM EGTA, 0.1% Triton X-100, 0.3 M sucrose, 1 mM dithiothreitol, 0.1 mM ATP, 1 mM Na_3_VO_4_ and 10 mM NaF supplemented with protease and phosphatase inhibitors (Roche Diagnostics, Indianapolis, IN, USA). Cell lysates were scraped and collected into microfuge tubes and allowed for 10 min lysis on ice. For extracting chromatin-bound proteins, the detergent-insoluble nuclei were separated from the soluble fraction by centrifugation at 3000*g* for 5 min. The soluble fractions were transferred to new microfuge tubes, and the remaining pellets were washed twice with CSK buffer. Protein concentrations were measured with Bio–Rad Protein Assay Dye Reagent Concentration (Bio–Rad, Hercules, CA, USA) at 595 nm and aliquoted for mixing with Laemmeli sample buffer (Bio–Rad) at equivalent protein contents. Protein denaturation was performed at 95 °C for 10 min. Denatured samples were resolved by SDS (sodium dodecyl sulfate)-PAGE (polyacrylamide gel electrophoresis) gels and transferred to nitrocellulose membranes by electrophoresis and Trans-Blot Turbo systems (Bio–Rad). After transferring to nitrocellulose membranes and blocking with 5% nonfat milk, the targeted proteins were immunoblotted with specific antibodies. To probe different target proteins on the same membranes, a stripping buffer containing 62.5 mM Tris–HCl (pH 6.8), 2% SDS and 0.1 M β-mercaptoethanol was used to strip the primary and secondary antibodies.

### 2.6. Immunoprecipitation Assay

For endogenous protein interaction, cells were treated with MG132 (20 μM) for five hours before harvesting. After protein extraction and SDS-PAGE electrophoresis, cell lysates of the soluble fraction extracted with CSK buffer were aliquoted equally for incubating with goat serum- or goat anti-BCA2 antibody-conjugated protein A/G plus agarose (Santa Cruz Biotechnology) overnight at 4 °C, separately. The conjugation of the antibody to protein A/G agarose was conducted by adding and mixing 4 μg BCA2 antibody and 50 μL protein A/G agarose with 1 mL immunoprecipitation (IP) buffer (25 mM Tris–HCl pH 7.4, 150 mM NaCl, 1 mM EDTA, 1% NP-40 and 5% glycerol) by rotation for two hours at 4 °C. The excessive non-conjugated antibody was removed by washing in the IP buffer with 30 s centrifugation at 12,000 rpm for three times.

For cell lysates acquired from the chromatin-bound fraction, DNA digestion was processed with RNase-free DNAase I in CSK buffer for 45 min at 37 °C, following the manufacturer’s protocol (Fisher Scientific, Pittsburgh, PA, USA) prior to the incubation with goat serum- or goat anti-BCA2 antibody-conjugated protein A/G plus agarose. To solubilize chromatin-bound proteins, DNAase I-processed samples were further mixed with EDTA at the final concentration of 4.5 mM and heated at 65 °C for 10 min. Afterwards, samples were centrifuged at 12,000 rpm for two minutes to fractionate solubilized chromatin-bound proteins in the supernatant. After overnight incubation with goat serum and goat anti-BCA2 antibody-conjugated protein A/G plus agarose, IP samples were washed with IP buffer by 30 s centrifugation at 12,000 rpm three times. The precipitated proteins then were eluted by dissolving in 30% (*w*/*v*) sarkosyl-containing IP buffer with 30 min rotation at 4 °C, followed by 30 s centrifugation at 12,000 rpm. The supernatants were collected and boiled for 10 min with Laemmeli sample buffer, and subject to SDS-PAGE electrophoresis.

### 2.7. Quantitative Reverse Transcription-Polymerase Chain Reaction

The mRNA levels of BCA2, ATM, and Rad51 were examined by two-step PCR (polymerase chain reaction) in MCF-7 cells. After cell treatment or transfection by siBCA2, total RNAs were prepared from cultured cells using TRIzol reagent (Ambion; Life Technologies). Reverse transcription of 1 μg of total RNA was performed with the High Capacity RNA-to-cDNA Kit (Applied Biosystems; Life Technologies) following the manufacturer’s protocol. The primers for real-time PCR reaction were acquired from Bio–Rad and were compatible with the iTaq Universal SYBR Green Supermix (Bio–Rad) and CFX96-Real-Time System (C1000 Touch Thermal Cycler, Bio–Rad). PCR on cDNA products was performed using the following parameters: pre-denaturation at 95 °C for 30 s, denature at 95 °C for 5 s, annealing and elongation at 60 °C for 30 s, and running 50 cycles with a final melt-curve analysis set from 65 to 95 °C at a 0.5 °C increment, 2–5 s/step. The folds of changes in target mRNA levels were calculated from the *C*_t_ (cycle threshold) values of samples in triplicate.

### 2.8. Clonogenic Assay

Cells undergoing log-phase growth were plated at the density of 1000 cells/6 cm dish. Following cell attachment at 4 h post-seeding, treatment was carried out and terminated by replacing the treatment medium with growth media. Following two weeks of culture, *i.e.*, until the number of colonies per dish did not increase with culture time, the cells were washed with PBS and fixed with 100% methanol for 10 min. The colonies were stained with crystal violet (0.2% crystal violet (*w*/*v*) in water) for 5 min, and rinsed with PBS. Colonies, composed of 50 cells and more, were counted in control and treated dishes and used to calculate the surviving fraction of cells receiving treatment.

### 2.9. Cell Cycle Analysis with Fluorescent-Activated Cell Sorting (FACS)

Cells at 60% confluence were harvested after treatment. The cells were trypsinized, washed, and fixed with ice-cold 70% ethanol at −20°C overnight. After PBS washing, cells were incubated with 40 µg/mL propidium iodide (Life Technologies) and 0.5 µg/mL RNase A (Thermo Scientific) in PBS at 37 °C for 30 min, and subjected to flow cytometry performed with a BD FACSCanto II system (Beckton–Dickinson, Franklin Lakes, NJ, USA) and FACS Diva v6.1.3 software (BD Biosciences, San Jose, CA, USA). Cell cycle distribution was analyzed using the trapezoid shape approximation of ModFit (Verity Software House, Inc., Topsham, ME, USA) with linear scaling.

### 2.10. Immunofluorescence Staining and Confocal Microscopy

Cells were seeded in fluoroDish culture plates (World Precision Instruments, Inc., Sarasota, FL, USA) and treated with Erb-041 and/or UV at 70% confluence. After irradiation, cells were incubated with EdU (5-ethynyl-2'-deoxyuridine), an indicator for DNA synthesis, for 30 min and fixed with acetone–methanol, 1:1 (*v*/*v*) for 10 min at −20 °C. Following fixation, the samples were washed in PBS for 5 min and blocked with 5% milk TBST solution (20 mM Tris base, 0.15 M NaCl and 0.05% Tween 20) for 30 min at room temperature. EdU staining was performed following the manufacturer’s protocol (Click-iT EdU Alexa Fluor 647 Imaging Kit, Life Technologies). Further incubation of samples with the primary antibody against γH2AX was performed for 4 hours after washing with 3% BSA in PBS. After washing away the unbound primary antibody with PBS three times, cells were incubated with a fluorophore-tagged secondary antibody for one hour, and washed with PBS three times at room temperature prior to nuclear staining with ProLong Gold antifade reagent with DAPI (Life Technologies, Eugene, OR, USA). For the immunofluorescence staining of MCF-7 cells with BCA2 and Rad51 primary antibodies, cells were fixed with acetone–methanol, 1:1 (*v*/*v*) prior to the incubation with antibodies and the nuclear staining. A Nikon A1rsi laser scanning confocal microscope with a Plan Apo λ 100X/NA 1.45 oil immersion objective was utilized for imaging the localization and expression of EdU and γH2AX as indicated by the secondary antibodies Alexa Fluor 647 anti-EdU and Alexa Fluor 488 anti-mouse IgG, respectively. The localization and expression of Rad51 and BCA2 were determined based on the excitation signals from Alexa Fluor 488 anti-rabbit and Alexa Fluor 555 anti-goat IgG, along with DAPI excitation wavelength at 405 nm.

### 2.11. Statistical Analysis

The data from the clonogenic assay and RT-PCR were normalized to the control groups. The statistical significance was tested by multiple comparisons with the Bonferroni correction, using the ProStat statistical software package (Polysoftware, Pearl River, NY, USA). Error bars indicate standard deviations of triplicate runs.

## 3. Results

### 3.1. Erb-041 Decreased Estrogen Receptor α (ERα) Signaling While Inhibiting Breast Cancer Associated-Gene 2 (BCA2) Transcription

**Figure 1:**
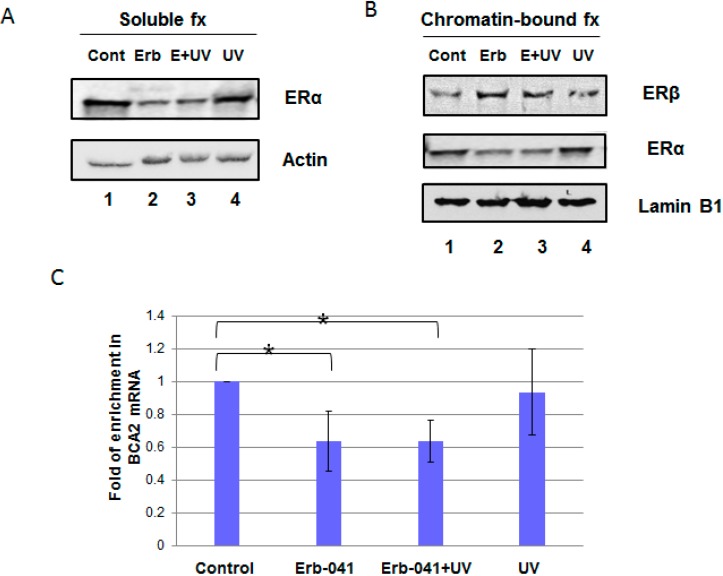
Erb-041 suppressed UVC-induced chromatin association of ERα in response to ERβ upregulation. (**A**) MCF-7 cells were treated with Erb-041 for 24 h prior to irradiation, as indicated in panels (**A**–**C**). Cell lysates from exponentially growing MCF-7 cells were fractionated into chromatin-bound (**B**) and soluble (**A**) fractions. The samples were subjected to SDS-PAGE electrophoresis and immunoblotting using anti-ERα, ERβ, actin, lamin B1 and β-tubulin antibodies. The latter two blots were set as internal controls for the proteins expressed in the chromatin-bound and soluble fractions; (**C**) The mRNA levels of BCA2 were analyzed using real-time RT-PCR. GAPDH was used as a loading control. The error bars represent standard deviations in this figure, as well as in all the other graphical figures. * denotes significant difference between control and experimental groups with *p* < 0.05 (*n* = 3 for all measurements). Description of experimental conditions: lanes 1, cells without any treatment (denoted by Cont); lanes 2, cells incubated with 40 μg/mL Erb-041 for 24 h (denoted by Erb); lanes 3, cells incubated with 40 μg/mL Erb-041 for 24 h before UVC irradiation at the energy irradiance of 10 J/m^2^ (denoted by E+UV); lanes 4, cells irradiated by 10 J/m^2^ UVC (denoted by UV).

To characterize the effect of Erb-041 on ERα-mediated signaling in MCF-7 cells, we separated cell lysates into soluble and chromatin-bound fractions for protein analysis (Figure 1A,B). We found that 24 h incubation with Erb-041 not only decreased chromatin-bound ERα (Figure 1B, lane 2 *vs.* lane 1) but also diminished UVC-induced chromatin association of ERα in MCF-7 cells (Figure 1B, lane 3 *vs.* lane 4). In contrast, chromatin-bound ERβ was increased by Erb-041 in the absence or presence of radiation (Figure 1B, lanes 2 and 3 *vs.* lane 1). Concurrently, the levels of BCA2 transcripts were decreased by Erb-041, suggesting the ERβ agonistic effect of Erb-041 antagonizes ERα-mediated BCA2 transcription (Figure 1C). These results indicate that Erb-041 reduces ERα chromatin association and BCA2 transcription.

### 3.2. Erb-041 Induced the Accumulation of Cells in S Phase and Potentiated DNA Synthesis, Reducing the Repair of Ultraviolet-Induced DNA Damage

**Figure 2:**
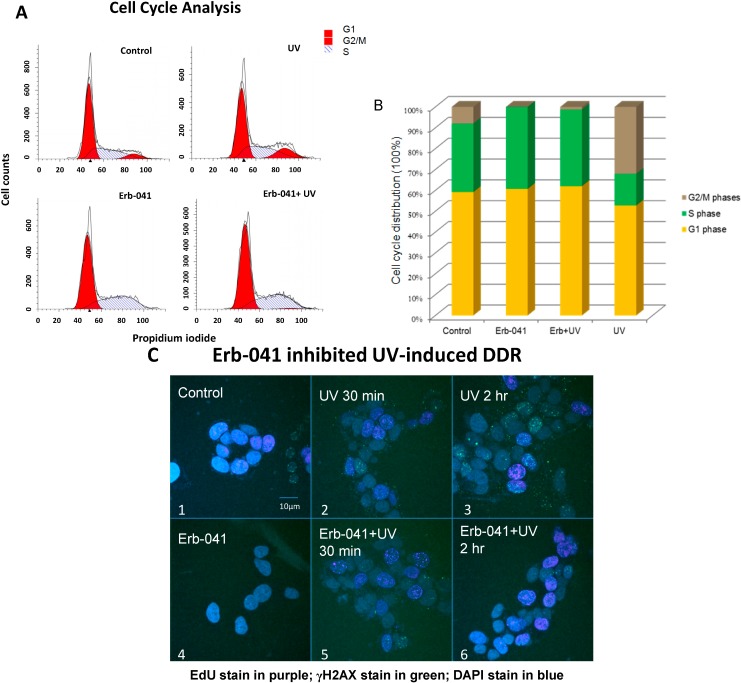
Erb-041 mediated cell cycle arrest in S phase and potentiated UV-induced DNA synthesis (**A**) MCF-7 cells were treated with Erb-041 for 24 h prior to UVC irradiation. After irradiation, exponentially growing cells were further incubated for 24 h prior to overnight ethanol fixation. The amount of DNA in each cell was measured by FACS after propidium iodide staining; (**B**) Quantitative analysis of the cell cycle distribution in the setting described in (**A**); (**C**) DNA synthesis and damage were assayed by immunofluorescence staining with the nucleotide analog, EdU, as well as anti-γH2AX antibody. After being treated with Erb-041 for 24 h, cells continued to undergo 30 min EdU incorporation, which was terminated by PBS wash and medium replacement. Cell irradiation was then carried out prior to fixation and subsequent incubation with anti-EdU reaction buffer, anti-γH2AX antibody, the designated fluorophore antibody and DAPI. EdU incorporation was represented by the purple fluorescence of Alexa Fluor 647 while γH2AX foci and the cell nuclei were indicated by the green fluorescence of Alexa Fluor 488 and DAPI, respectively. Description of experimental conditions: image #1, cells without any treatment; image #2, cells irradiated by 10J/m^2^ UVC and incubated for 30 min after irradiation; image #3, cells irradiated by 10J/m^2^ UVC and incubated for 2 h after irradiation; image #4, cells incubated with 40 μg/mL Erb-041 for 24 h; image #5, cells incubated with 40 μg/mL Erb-041 for 24 h before the UVC irradiation and 30 min post-irradiation incubation; image #6, cells incubated with 40 μg/mL Erb-041 for 24 h before the UVC irradiation and two-hour post-irradiation incubation.

Next, we checked the cell cycle distribution (Figure 2A,B) as well as cell proliferation and DNA damage response (Figure 2C). UVC-induced G2/M accumulation at 24 h post-irradiation was compromised by the arrest and accumulation of cells in S phase upon Erb-041 pretreatment. Moreover, the EdU incorporation assay indicates that DNA synthesis decreased with time in the non-irradiated group while DNA synthesis in the irradiated group increased (Figure 2C, photos 1–3 *vs.* photos 4–6). Intriguingly, the number of γH2AX foci preferentially increased with time in the irradiated group compared with the non-irradiated group. This result suggests that pretreatment with Erb-041 arrests cells in S phase and compromises the intra-S-phase DNA damage checkpoint evoked by UVC, leading to fewer DNA double-stranded breaks generated from post-replication recombination repair.

### 3.3. Erb-041 Exacerbated UV-induced DNA Damage through Reduction of BCA2 Expression and Mitigation of DNA Damage Response

**Figure 3:**
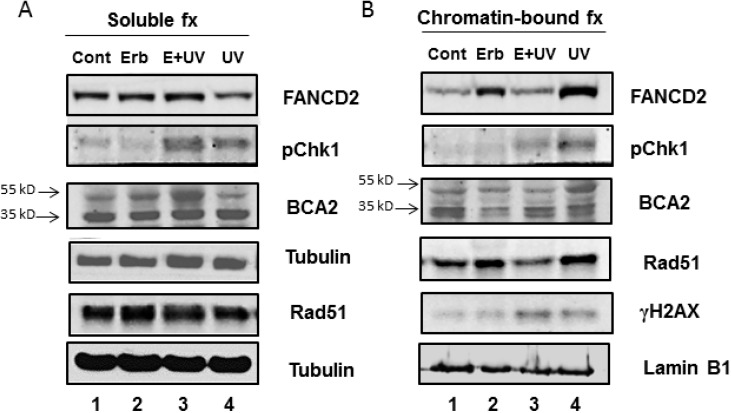
Erb-041 inhibited the chromatin association of BCA2 and reduced UV-induced DDR protein expression. After Erb-041 pretreatment or/and irradiation, exponentially growing MCF-7 cells were lysed and the cell lysates were fractionated and subjected to SDS-PAGE and immunoblotting with the indicated antibodies. Description of experimental conditions: Lanes 1, cells without any treatment (denoted by Cont); lanes 2, cells incubated with 40 μg/mL Erb-041 for 24 h (denoted by Erb-041); lanes 3, cells incubated with 40 μg/mL Erb-041 for 24 h before UVC irradiation at the energy irradiance of 10 J/m^2^ (denoted by E+UV); lanes 4, cells irradiated by 10J/m^2^ UVC (denoted by UV). Protein molecular weights at 35 and 55 KD indicate the non-ubiquitinated and polyubiquitinated BCA2.

In regard to Erb-041-induced S-phase cell population, the S phase-specific DNA repair proteins including FANCD2 and Rad51 were upregulated in the chromatin-bound fractions without evoking Chk1 and H2AX phosphorylation (Figure 3B, lane 2 *vs.* lane 1). In contrast, chromatin-bound BCA2 was decreased by Erb-041 in both its ubiquitinated and non-ubiquitinated forms (Figure 3B, lane 2 *vs.* lane 1). When cells were exposed to UVC, BCA2 was highly ubiquitinated and stably associated with chromatin, while phospho-Chk1, FANCD2 and Rad51 were also increased in association with chromatin in response to UV-induced DNA damage (Figure 3B, lane 4 *vs.* lane 1). However, DDR was diminished by pretreatment with Erb-041, leading to more diffused γH2AX, indicative of unresolved and unchecked DNA damage (Figure 3B, lane 3 *vs.* lane 4). Upon Erb-041 pretreatment, UVC-induced BCA2 ubiquitination was destabilized as ubiquitinated BCA2 dissociated from chromatin and was retained in the soluble fraction (Figure 3A,B, lanes 3 *vs.* lanes 4). Along with BCA2 solubilization, FANCD2, phospho-Chk1 and Rad51 (Figure 3A,B, lanes 3 *vs.* lanes 4) were also solubilized upon Erb-041 pretreatment. This result indicates that BCA2 may play a role in mediating Erb-041- modulated DNA damage response and repair.

### 3.4. BCA2 Knockdown Intrinsically Induced DNA Damage Response and Impeded the Repair of UV-Induced DNA Damage

**Figure 4:**
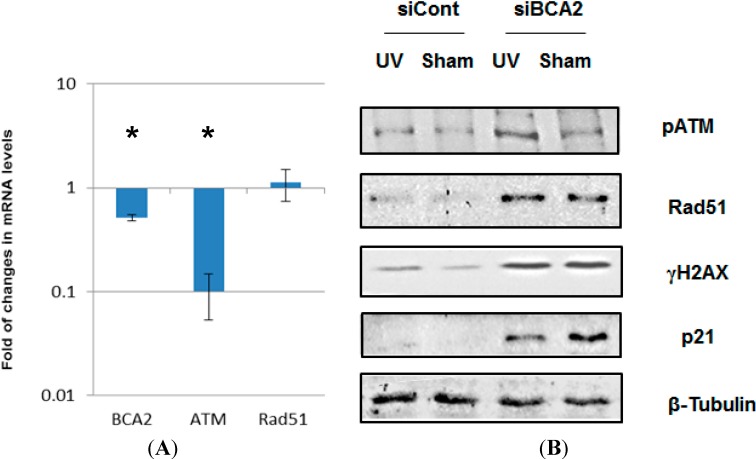
*BCA2* knockdown up-regulated several DDR proteins. (**A**) Transcriptional levels of BCA2, ATM and Rad51 genes were analyzed with quantitative real-time PCR at 72 h post-transfection with scrambled or BCA2-targeted siRNAs; (**B**) Replicate cultures of MCF-7 cells were transfected with siRNA against BCA2 or non-targeting control siRNA prior to irradiation and post-irradiation incubation. At 2 h post-irradiation, cell extracts were isolated and analyzed by SDS-PAGE and immunoblotting with the indicated antibodies. * denotes significant difference between control siRNA- and BCA2 siRNA-treated groups with *p* < 0.05.

To investigate the effect of BCA2 on DNA damage response and repair, BCA2-targeted siRNA was used to specifically knock down BCA2 (Figure 4A). Interestingly, the down-regulation of BCA2 transcripts through siRNA transfection was accompanied by a significant decrease in ATM mRNA. On the other hand, DDR proteins, phospho-^1981^Ser-ATM, Rad51 and γH2AX, were increased along with a substrate of BCA2 ubiquitin ligase, p21, in MCF-7 cells upon BCA2 knockdown (Figure 4B). With respect to the insignificant up-regulation of Rad51 mRNA detected after BCA2 knockdown, BCA2 was hypothesized to regulate phospho-^1981^Ser-ATM and Rad51 not at the transcriptional level but at the posttranslational level. These results indicate that BCA2 may be essential for maintaining genomic stability via regulation of the turnover rates of DDR proteins and cell cycle checkpoint.

### 3.5. BCA2 Endogenously Interacted with γH2AX and Rad51 in Association with Ubiquitin-Mediated Degradation

**Figure 5:**
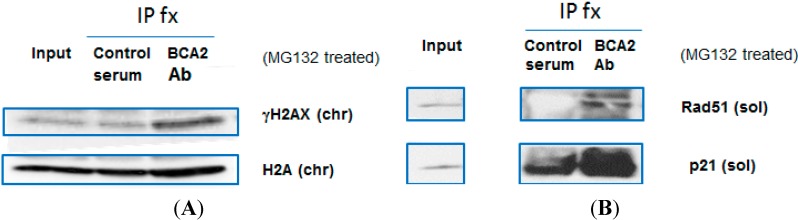
BCA2 interacts with **γ**H2AX and Rad51. (**A**,**B**) MCF-7 cells were exposed to 20 μM MG132 for 5 h and collected for immunoprecipitation using goat serum or anti-goat BCA2 antibody along with Protein A/G PLUS Agarose IP Reagent. The precipitated proteins were fractionated and subject to SDS-PAGE and immunoblotting with the indicated antibodies. Description of experimental conditions: chr, the chromatin-bound fraction of cell lysates; sol, the soluble fraction of cell lysates.

In regard to the fact that BCA2 contains a RING-finger domain that interacts and ubiquitinates substrates [16,28], we further investigated whether BCA2 participated in DDR via its interaction with proteins involved in DNA damage repair. MCF-7 cells were treated with the proteasome inhibitor, MG132, for five hours prior to immunoprecipitation assays (Figure 5A,B; right IP lanes *vs.* left IP lanes). The endogenous BCA2 was fractionated and immunoprecipitated by the anti-BCA2 antibody, and the endogenous γH2AX and Rad51, along with the known substrate, p21, were co-immunoprecipitated (Figure 5A,B). Without marked, detectable upregulation of non-phosphorylated H2A, this result suggests that γH2AX could be a specific substrate for BCA2 in addition to Rad51 in the process of maintaining genome integrity and stability (Figure 5A).

### 3.6. Rad51 Protein Degradation May Be Preceded by the Nuclear Co-Localization of BCA2 and Rad51 Under Co-Treatment with Erb-041 and UVC

**Figure 6:**
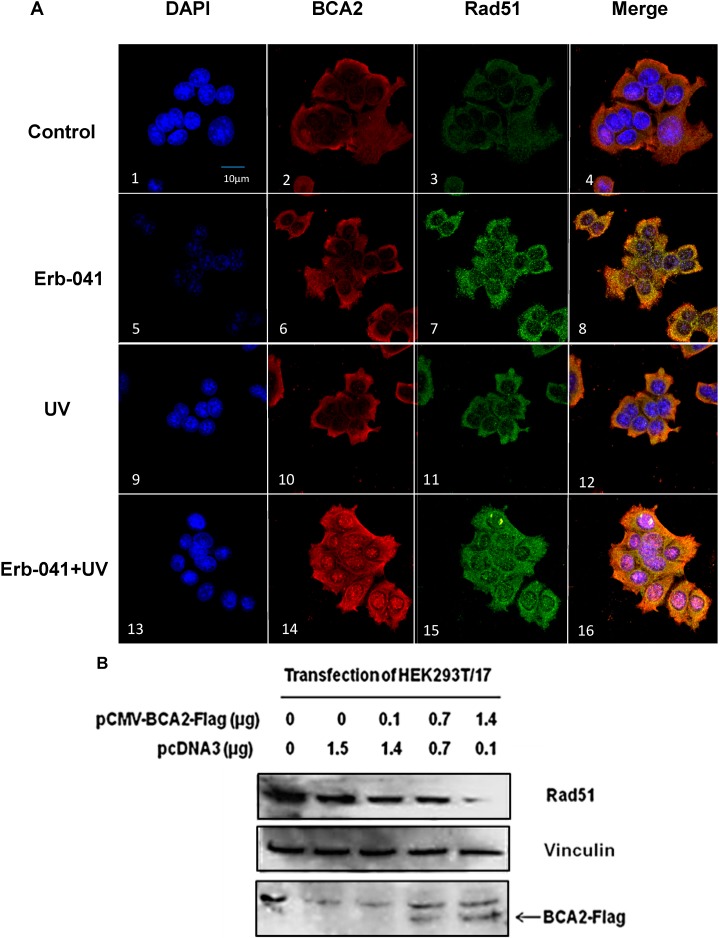
Erb-041 potentiated BCA2 and Rad51 nuclear co-localization in association with ubiquitin-mediated Rad51 degradation. (**A**) MCF-7 cells under different experimental conditions were fixed and immunostained with anti-BCA2 and Rad51 antibodies prior the staining with Alexa Fluor 488 anti-goat, Alexa Fluor 555 anti-rabbit IgG, and the nuclear stain, DAPI, for confocal microscopic imaging; (**B**) The pCMV-BCA2-Flag or pcDNA3 vector was transfected into HEK293T/17 cells. The cells were lysed 48 hours later, and the cell lysates were subject to SDS-PAGE and immunoblotting with anti-Rad51, actin and FLAG antibodies. Description of experimental conditions: Control, cells without any treatment; Erb-041, cells incubated with 40 μg/mL Erb-041 for 24 h; UV, cells irradiated by 10 J/m^2^ UVC; Erb-041+UV, cells incubated with 40 μg/mL Erb-041 for 24 h before UVC irradiation at the energy irradiance of 10J/m^2^; first-lane images, DAPI immunofluorescence under different experimental conditions; second-lane images, anti-BCA2 immunofluorescence under different experimental conditions; third-lane images, anti-Rad51 immunofluorescence under different experimental conditions; fourth-lane images, merged immunofluorescence under the different experimental conditions.

The effect of Erb-041 on BCA2 interaction with Rad51 was assessed by immunofluorescence staining (Figure 6A). Based on the confocal microscope images, Erb-041 increased the expression of Rad51 (Figure 6A, image 7 *vs.* image 3), and subsequent irradiation resulted in the strong nuclear co-localization of BCA2 and Rad51 (Figure 6A, image 16 *vs.* image 8). Combined with the observation of Erb-041-mediated dissociation of BCA2 from chromatin (Figure 3A,B, lanes 3), this result indicates that Erb-041 impaired DDR partially via BCA2 interaction with Rad51 and the concurrent dissociation from chromatin. To study the consequence of BCA2 interaction with Rad51, we transfected HEK293T/17 cells with a pCMV-BCA2-Flag construct in the absence of MG132 (Figure 6B). When recombinant BCA2 was gradually overexpressed in HEK293T/17 cells, the levels of endogenous Rad51 decreased in a BCA2 overexpression-dependent manner. This suggests that Rad51 may be targeted for ubiquitin-mediated degradation by BCA2, leading to compromised DNA repair via the HR pathway.

## 4. Discussion

About a decade ago, researchers found that the prognosis for patients with ER-positive invasive breast cancers was better than that for patients with ER-negative non-aggressive cancers. The difference in prognosis correlated with BCA2 over-expression, which alternatively promotes the cell death of ER-positive breast cancers in association with functional DNA damage checkpoints, leading to negative regional recurrence of breast cancers [11]. In the present study, we have identified the role of BCA2 in maintaining the genomic integrity of ER-positive MCF-7 cells, and revealed a mechanism by which an ERβ agonist compromised BCA2-mediated DNA damage response and repair. It was found that Erb-041 inhibited chromatin association of ERα, leading to the reduced expression of BCA2. In support of our observation, ERα has been reported to bind to the estrogen response element of BCA2 and promote the gene transcription of p21 [12,29]. Through the association with cyclin−cdk complexes, p21 mediates cell cycle checkpoints at G1/S and G2/M transitions, thus permitting DNA repair to take place [30,31]. As ubiquitination of p21 by BCA2 promotes p21 proteosomal degradation, the stabilization of chromatin-bound BCA2 following UV irradiation was hypothesized to facilitate the activation of DNA damage checkpoints (because of free/unbound nuclear p21 to halt the cell cycle) [13]. Correspondingly, UV-induced lethal DNA damage was increased by Erb-041 pretreatment along with downregulation of chromatin-bound BCA2, suggesting that Erb-041 may potentiate UV-induced DNA damage via abrogation of cell cycle checkpoints. As expected, DNA synthesis in Erb-041-pretreated cells was less impeded by UV irradiation, compared to non-treated cells.

Erb-041-mediated chromatin dissociation of BCA2 attenuated the cells’ DNA damage response and repair in intra S phase. Similar to BCA2 silencing, Erb-041 increased the expression of Rad51 and FANCD2 in the cells’ chromatin-bound fraction. When cells are incur to UV damage during DNA synthesis, the UV-induced oxidative DNA adducts can be converted to DNA DSBs during replication [32]. Some replication-coupled recombination repair pathways, such as the Fanconi Anemia pathway and homologous recombination are thus activated to repair post-replication DSBs by protein modification, including phosphorylation and ubiquitination [33,34]. Our results show that two crucial proteins in the FA and HR pathways, *i.e.*, FANCD2 and Rad51, were decreased by Erb-041 pretreatment in the chromatin-bound fraction of MCF-7 cell lysates upon UV irradiation. Consistent with the regulation of DDR protein expression, the distribution of γH2AX becomes increasingly diffuse, following Erb-041 pretreatment plus irradiation, compared with irradiation alone.

Interestingly, BCA2 silencing intrinsically increased γH2AX, Rad51 and phospho-ATM, suggesting the involvement of BCA2 in DNA damage response and repair presumably via ubiquitination. During the process of DNA repair, histone H2AX is often monoubiquitinated by RING-finger proteins and phosphorylated by ATM, thus recruiting DDR proteins to chromatin for repairing DNA double-stranded breaks [35,36,37]. Our results demonstrated that BCA2 can interact with Rad51 and γH2AX, presumably leading to Rad51 co-localization with the sites of double-stranded breaks. Moreover, the interaction of BCA2 and Rad51 highlights the role of BCA2 in maintaining genomic stability during *de novo* synthesis. On the contrary, Erb-041-induced BCA2 solubilization from chromatin is expected to accelerate Rad51 degradation upon BCA2-mediated ubiquitination. To summarize, this study describes a novel mechanism for treating ER-positive breast cancers using Erb-041 or drugs that share similar functions.

**Figure 7:**
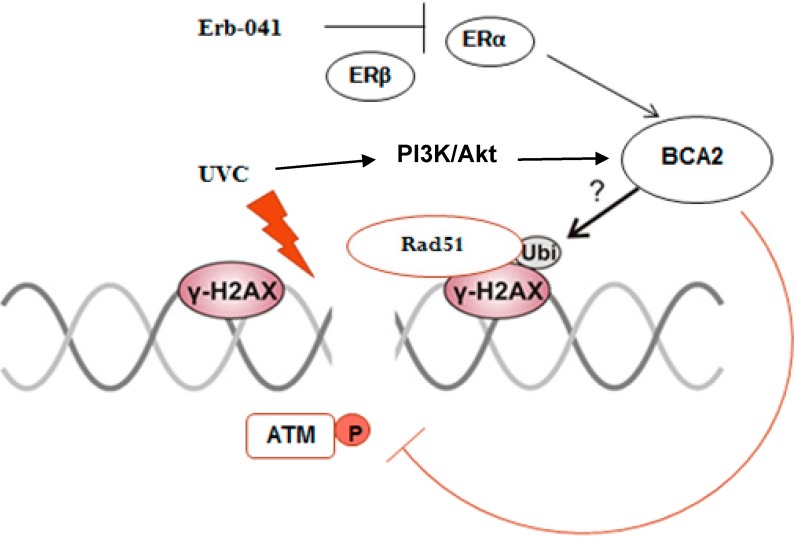
Proposed working model. In response to UVC, MCF-7 cells increase the ubiquitination activity and chromatin-binding ability of BCA2 through AKT-mediated BCA2 activation [28,38]. The association of BCA2 with γH2AX incites a BCA2/H2AX/ATM axis, initiating the DDR and controlling breast cancer cell sensitivity. The importance of Erb-041 in suppressing DNA recombination repair through BCA2 inhibition is based on the mitigatory effect of Erb-041 on AKT activation and ERα-mediated BCA2 transcription [4]. By pretreating MCF-7 cells with Erb-041, UV-induced chromatin-bound Rad51 and BCA2 became solubilized, leading to the abolition of DNA repair.

Taken together, the present study shows the mechanism by which UV-induced DNA damage response and repair can be inhibited by an ERβ agonist that has been linked to ERα-dependent signaling. As illustrated in Figure 7 (the Proposed Working Model), the basal level of ERα promotes DNA damage response and repair, thus maintaining cell survival after irradiation by stabilizing the chromatin association of Rad51 and BCA2. With Erb-041 pretreatment, UV-induced DNA damage response and repair are presumably compromised by the failure to ubiquitinate H2AX and a consequent decrease in Rad51 chromatin association. This study suggests a role of ER/BCA2 signaling in facilitating DNA repair and the potential for targeting BCA2 to sensitize ER-positive cells to oxidative DNA damage induced by UV, X-ray radiation, or chemotherapeutic agents.

## 5. Conclusions

Estrogen receptors have long been targeted for breast cancer treatment. Anti-estrogenic drugs have been reported to synergize the growth inhibitory effect of ionizing radiation [39]. The Erb-041-mediated reduction of chromatin-bound BCA2 increased the levels of chromatin-bound γH2AX upon UVC irradiation, indicating an important role of BCA2 in regulating DNA damage response and repair. In response to estrogen-induced oxidative DNA damage, ER-positive MCF-7 cells timely initiated γH2AX foci formation in the presence of an inherently corresponding effector, BCA2, indicating its important role in eliminating DNA damage and promoting cell proliferation [40]. In this study, we demonstrated that Rad51 and γH2AX interact with BCA2 in correlation with the chromatin-binding capability of BCA2. BCA2 knockdown increased the number of DSBs, ATM phosphorylation, and the expression of proteins involved in DNA homologous recombination repair. Treatment with UVC also resulted in a large number of DNA DSBs in MCF-7 cells under *BCA2* knockdown. This indicates a role of BCA2 in maintaining genomic stability and mediating DDR towards endogenous and external genotoxic stress, such as replication-/transcription-coupled and chemical-induced DNA DSBs. Disregarding the cancer type and background, including ATM, BRCA2 and p53 mutations or PTEN (phosphatase and tensin homolog) loss, the inhibition of BCA2 by either anti-ERα endocrine therapy or anti-BCA2 gene therapy holds promise for the neoadjuvant treatment of breast cancers as most of them possess functional BCA2.

## Abbreviations

BCA2: breast cancer-associated gene 2
DDR: DNA damage response
DSB: double-stranded break
ER: estrogen receptor
ERα: estrogen receptor α
HR: homologous recombination
